# Supplementary material for: EcDBS1R4, an Antimicrobial Peptide Effective against Escherichia coli with In Vitro Fusogenic Ability
Source: Int J Mol Sci. 2020 Nov 30;21(23):9104. doi: 10.3390/ijms21239104 (PMC7730630; doi:10.3390/ijms21239104)
Supplement: Supplementary file 1 [file ijms-21-09104-s001.pdf]

Supporting Information

EcDBS1R4 an antimicrobial peptide with *in vitro* fusogenic ability selective to *Escherichia coli*

Marcin Makowski[1], Mário R. Felício[1], Isabel C. M. Fensterseifer[2,3], Octávio L. Franco[2,3,4], Nuno C. Santos[1#], Sónia Gonçalves[1#]

[1]Instituto de Medicina Molecular, Faculdade de Medicina, Universidade de Lisboa, Lisbon, Portugal.

[2]Centro de Análises Proteômicas e Bioquímicas, Pós-graduação em Ciências Genômicas e Biotecnologia, Universidade Católica de Brasília, Brasília, DF, Brazil.

[3]Programa de Pós-Graduação em Patologia Molecular, Universidade de Brasília, Brasília, DF, Brazil.

[4]S-Inova Biotech, Pós-graduação em Biotecnologia, Universidade Católica Dom Bosco, Campo Grande, MS, Brazil.

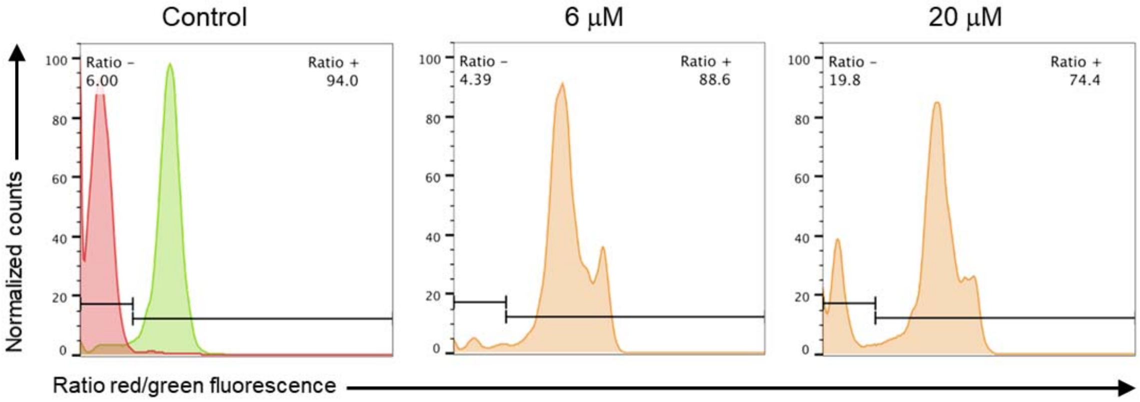

Figure S1. Membrane surface potential studies of *E. coli* exposed to EcDBS1R4. Histograms of the fluorescence ratio obtained by flow cytometry for *Escherichia coli* (ATCC 25922) with 0, 6 and 20 μM of EcDBS1R4. For staining, we used 15 μM of 3,3'-diethyloxacarbocyanine iodide ($DiOC_2(3)$), a green dye that accumulates on hyperpolarized membranes (green histogram), but that is red-shifted as the dye self-associates under larger membrane potentials[1]. 10 μM of the proton ionophore carbonyl cyanide 3-chlorophenylhydrazone (CCCP) was used as a control of total depolarization (red histogram).

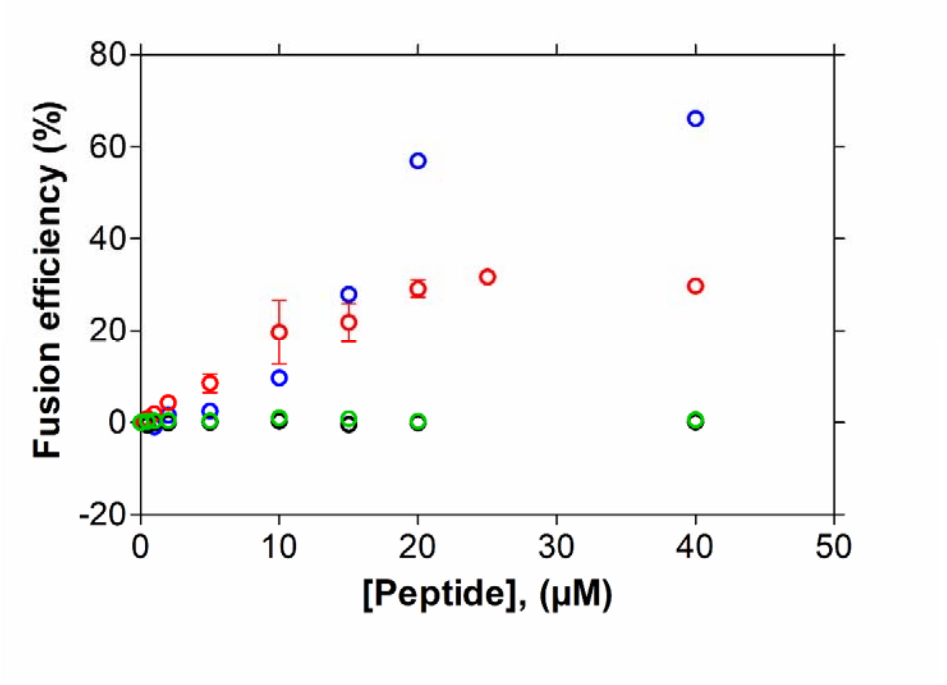

Figure S2. Fusion / hemifusion efficiency of CL-rich lipid vesicles promoted by titration with EcDBS1R4. Percentage of fusion efficiency was calculated using equation 8. Lipid vesicles used were POPC (black), POPC:Chol (70:30) (green), POPG:CL (80:20) (blue) and POPE:POPG:CL (20:60:20) (red).

Table S1. ***In silico*** **predicted interactions between EcDBS1R4 and anionic/zwitterionic mimetic membranes**[2].

**Membrane**	**Residue number**	**Peptide**			**Lipid**			**Distance, Å**
		**Residue**	**Position**	**Atom**	**Type**	**Position**	**Atom**	
**POPC**	1	Lys	4	NZ	POPC	15	O14	2.7
	2	Arg	9	NH1	POPC	4	O13	3.0
	3	Val	15	CG2	POPC	2	C15	3.4
	4	Val	15	CG1	POPC	3	C13	3.5
	5	Trp	19	NE1	POPC	11	O13	3.2
	6	Trp	19	NE1	POPC	11	O14	3.3
**POPC:Chol (70:30)**	1	Lys	5	NZ	Chol	1	O3	3.1
	2	Arg	9	NH1	POPC	1	O22	3.0
	3	Lys	13	NZ	POPC	1	O13	3.0
**POPC:POPG (70:30)**	1	Met	2	SD	POPC	4	O13	3.5
	2	Lys	4	NZ	POPC	12	O14	3.0
	3	Ala	8	O	POPG	1	OC2	3.3
	4	Ala	8	CB	POPC	4	C15	3.7
	5	Arg	9	NH2	POPG	3	OC2	3.1
	6	Arg	9	NH1	POPC	10	O22	2.9
	7	Arg	9	NH1	POPC	10	O14	3.3
	8	Val	15	O	POPC	2	O12	3.7
	9	Val	15	CG2	POPC	6	C13	3.7
	10	Ala	16	CB	POPC	2	C13	3.0
	11	Trp	19	NE1	POPC	9	O13	3.4

References

1. Domingues, M. M. *et al.* Antimicrobial protein rBPI21-induced surface changes on Gram-negative and Gram-positive bacteria. *Nanomedicine Nanotechnology, Biol. Med.* **10**, 543–551 (2014).

2. Cardoso, M. H. *et al.* A polyalanine peptide derived from polar fish with anti-infectious activities. *Sci. Rep.* **6**, 21385 (2016).
